# miR-106b-5p and miR-17-5p could predict recurrence and progression in breast ductal carcinoma in situ based on the transforming growth factor-beta pathway

**DOI:** 10.1007/s10549-019-05192-1

**Published:** 2019-04-15

**Authors:** Jieun Lee, Hee Eun Kim, Young-Seok Song, Eun Yoon Cho, Ahwon Lee

**Affiliations:** 1 Division of Medical Oncology, Department of Internal Medicine, College of Medicine, The Catholic University of Korea, Seoul, Korea; 2 Department of Hospital Pathology, College of Medicine, The Catholic University of Korea, 222 Banpo-daero, Seocho-gu, Seoul, 06591 Korea; 3 Cancer Research Institute, The Catholic University of Korea, Seoul, Korea; 4 Department of Pathology, Samsung Medical Center, Sungkyunkwan University School of Medicine, Seoul, Korea

**Keywords:** DCIS, Recurrence, miR-106b, miR-17, TGF-β

## Abstract

**Purpose:**

Ductal carcinoma in situ (DCIS) is well-known precursor of invasive ductal carcinoma (IDC). Parts of patients show recurrence as DCIS or IDC after local treatment, but there are no established markers predicting relapse. We analyzed changes in miRNA and oncogene expression during DCIS progression/evolution to identify potential markers predicting recurrence.

**Methods:**

Forty archival tissues diagnosed as primary or recurrent DCIS and DCIS adjacent to IDC were analyzed. MiRNA hierarchical clustering showed up-regulation of miR-17-5p and miR-106b-5p in recurrent DCIS and DCIS adjacent to IDC. Target genes were predicted based on pre-formed miRNA databases and PanCancer Pathway panel. MiRNAs were transfected into MCF-10A and MCF-7 cells; western blot analysis was performed with MCF-7 cell line to evaluate the effects on TGF-β downstream pathway.

**Results:**

miRNA hierarchical clustering showed 17 dysregulated miRNAs, including miR-17-5p and miR-106b-5p. Based on miRNA database and nCounter Pancancer pathway analysis, TGFβRII was selected as target of miR-106b-5p and miR-17-5p. MiR-106b-5p- and miR-17-5p-transfected MCF-7 cells showed decreased expression of TGFβRII, especially in cells transfected with both miRNAs.

**Conclusion:**

miR-106b-5p and miR-17-5p might have a role in breast cancer recurrence and progression by suppressing TGF-β activity, leading to early breast cancer carcinogenesis.

**Electronic supplementary material:**

The online version of this article (10.1007/s10549-019-05192-1) contains supplementary material, which is available to authorized users.

## Introduction

Ductal carcinoma in situ (DCIS) is a spectrum of pre-invasive lesions consisting of malignant cancer cells that accumulate within the intra-mammary lumen, without accompanying basement membrane invasion of the mammary duct [1]. DCIS is a precursor of invasive ductal carcinoma (IDC) based on the pathologic continuity of histologic findings [2] and similarity of gene expression profiles during the evolution of breast cancer cells [3].

The incidence of DCIS is increasing worldwide [4], accounting for 14% of all newly diagnosed breast cancer in Korea [5]. The current treatment of DCIS is based on surgical resection with a negative margin, followed by adjuvant radiation and hormonal therapy to prevent recurrence. However, approximately 10–20% of patients show local recurrence as DCIS or IDC during 15 years of follow-up [6–9]. Previous studies have reported various clinicopathologic features associated with recurrence as DCIS or IDC after surgical treatment. However, the pathologic, genetic, or molecular pathways contributing to the evolution of DCIS to IDC during recurrence remain elusive.

MicroRNAs (miRNAs) are non-coding small RNAs composed of 20–24 nucleotides. Alteration of miRNA expression is known to be closely associated with the pathogenesis of human malignancies [10]. Diverse miRNAs are specifically expressed in breast cancer, and alterations of miRNA expression levels are assumed to be associated with the recurrence or evolution of DCIS to IDC [11, 12]. However, no specific miRNAs have yet been identified as key factors contributing to the recurrence or progression of DCIS and IDC [12].

The transforming growth factor-beta (TGF-β) signaling pathway is associated with tumorigenesis of various malignancies, including breast, prostate, colorectal, and pancreatic cancer [13]. TGF-β has a paradoxical effect in breast cancer tumorigenesis; it acts as a tumor suppressor in benign breast epithelium, but shows tumor-promoting functions while breast tissues transform into malignant tissues [14]. The TGF-β pathway is partially regulated by miRNA clusters such as the miR-106b-25 and miR-17-92 [10, 15], in addition to various other miRNAs [16]. Previous studies analyzing the relationships between miRNAs and TGF-β pathway in breast cancer have mostly focused on highly invasive breast cancer, with little information available on their roles in low-invasive cell lines or premalignant breast tissues such as DCIS.

According to the low incidence of recurrent DCIS, the clinicopathologic characteristics of recurrent DCIS tend to be relatively underestimated. In this study, we analyzed the clinicopathologic characteristics and miRNA expression profiles of recurrent DCIS, comparing with primary DCIS and synchronous DCIS adjacent to IDC tissues. Furthermore, we selected highly dysregulated miRNAs among the recurrent DCIS tissue and studied their relationships to the TGF-β pathway with gene expression analysis of archival tissues and in vitro analysis of transfected breast cancer and normal breast cell lines.

## Materials and methods

### Patients

Between January 2000 and December 2010, we collected 40 samples diagnosed with primary DCIS, recurrent DCIS after primary treatment, or synchronous DCIS with IDC at Seoul St. Mary’s Hospital and Samsung Medical Center. Archival tumor tissues were reviewed by a senior pathologist for confirmation of pathologic parameters, including histologic grading, subtype, hormone receptor (HR) status, HER2 status, and to select areas of representative DCIS and IDC. HR-positive breast cancer was defined as ER or PR positive, HER2 negative by ASCO-CAP guideline [17, 18]; ER or PR positivity was defined as ≥ 1% tumor cells displaying nuclear staining. HER2 positivity was defined as HER2 immunohistochemistry 3 (circumferential membrane staining that is complete, intense, and within > 10% of tumor cells) or HER2 Silver in situ hybridization (SISH) positive (Dual-probe HER2/Chr17 ratio ≥ 2.0 or Dual-probe HER2/Chr17 ratio < 2.0 with an average HER2 copy number ≥ 6.0 signals/cell).

Patients who were diagnosed as DCIS and received breast-conserving surgery followed by adequate adjuvant hormonal, radiation therapy during follow-up period were assigned as group A (*n* = 10). Patients diagnosed as recurrent DCIS after breast-conserving surgery, adjuvant hormonal therapy, and radiation therapy were categorized as group B (*n* = 10). Regardless of operation method or adjuvant treatment during follow-up, patients who were diagnosed as pure DCIS (group C, *n* = 10) and synchronous DCIS with adjacent IDC (group D, *n* = 10) were selected.

This study was approved by the Institutional Review Board of Seoul St. Mary’s Hospital (KC14TIMI0933) and Samsung Medical Center (201510120001).

### RNA extraction

Each tumor was reviewed by a pathologist with specialty of breast pathology (A.L) on H&E stained slides. RNA was extracted from 5- to 10-μm-thick unstained tissue sections of formalin-fixed paraffin-embedded (FFPE) blocks using RNA extraction kit (Ambion, Austin, TX, USA). The tissue sections were manually microdissected to enrich for tumor cells and exclude inflammatory cells and stromal cells. For quality control, RNA purity and integrity were evaluated according to the absorbance ratio at 260/280 nm, and analyzed on the Agilent 2100 Bioanalyzer (Agilent Technologies, Palo Alto, USA).

### miRNA microarray profiling

Whole miRNA expression profiles of each sample were obtained using the Affymetrix Genechip miRNA 4.0 array process, according to the manufacturer’s protocols. RNA samples (200 ng) were labeled with the FlashTag™ Biotin RNA Labeling Kit (Genisphere, Hatfield, PA, USA). The labeled RNA was quantified, fractionated, and hybridized to the miRNA microarray according to the standard procedures provided by the manufacturer. The chips were washed and stained using a Genechip Fluidics Station 450 (Affymetrix, Santa Clara, CA USA). The chips were then scanned with an Affymetrix GCS 3000 scanner. Signal values were computed using the Affymetrix^®^ GeneChip™ Command Console software.

### Raw data preparation and statistical analysis of the microarray

Raw data were extracted automatically in the Affymetrix data extraction protocol using the Affymetrix GeneChip^®^ Command Console^®^ (AGCC) software. Importation of the CEL files, miRNA-level RMA + DABG-All analysis, and export of the results were conducted using Affymetrix^®^ Expression Console™ software. Array data were filtered by probe-annotated species.

The comparative analysis between the test sample and control sample was carried out according to the fc value and an independent t test, in which the null hypothesis was that no difference exists between the two groups. The false discovery rate was controlled by adjusting the *P* value using the Benjamini–Hochberg algorithm. The false discovery rate was set to < 0.05 and the fc level was set to ≥ 1.5, with a *P* value < 0.05. All statistical tests and visualization of differentially expressed genes were conducted using R statistical language v. 3.1.2.

### nCounter analysis

Using archival FFPE tissues, total RNA was re-extracted and analyzed using the Nanostring nCounter system (NanoString Technologies, Seattle, WA, USA). Based on the PanCancer Pathway panel (NanoString Technologies), the expression pattern of major genes involved in cancer biology were analyzed from the archival tissues.

### miRNA selection and target prediction

Hierarchical clustering of the miRNA microarray was performed for comparisons between groups A with B and between groups A with C. Commonly expressed dysregulated miRNAs in the group A–B cluster and group A–C cluster were selected and entered into three miRNA target databases (miRTarBase [http://mirtarbase.mbc.nctu.edu.tw/], miRsearch V3.0 [http://www.exiqon.com], and miRNAMap [http://mirnamap.mbc.nctu.edu.tw/]), and overlapping target genes were reviewed.

Estimated target genes based on miRNA target databases and major genes expressed based on the PanCancer Pathway panel were compared, and the final target genes were selected for the cell line experiments.

### Cell lines

The human breast cancer cell line MCF-7 was obtained from the Korean Cell Line Bank (Seoul, South Korea) and maintained in RPMI 1640 medium (Gibco BRL, Grand Island, NY, USA), supplemented with 10% fetal bovine serum (FBS) and 1% penicillin/streptomycin. The normal human breast cell line MCF10A was purchased from American Type Culture Collection (Rockville, MD, USA) and cultured in Dulbecco’s modified Eagle’s medium (Welgene, South Korea) supplemented with 10% FBS and 1% penicillin/streptomycin. Both cell lines were maintained at 37 °C in 5% CO_2_.

### miRNA mimics

The miRNA mimics and scrambled control miRNA used as a negative control were purchased from Genolution Pharmaceuticals (Seoul, South Korea). Sequences were as follows: 5′-CAAAGUGCUUACAGUGCAGGUAG-3′ (miR-17-5p mimic), 5′- UAAAGUGCUGACAGUGCAGAU-3′ (miR-106b-5p mimic), 5′-UUUUAACUCAGUAUUUUUA-3′ (scrambled control).

### RNA extraction, cDNA synthesis, and RT-qPCR using cell lines

MCF10A and MCF7 were harvested, and total RNA was extracted using TRIzol Reagent (Ambion, Carlsbad, CA, USA), according to the manufacturer’s instructions. The quality of RNA was confirmed using a Nanodrop ND-1000 spectrophotometer (Thermo Fisher Scientific, Waltham, MA, USA). cDNA was synthesized from 10 ng of total RNA using TaqMan microRNA Reverse Transcriptase kit (ABI, USA). The reaction conditions were as follows: 16 °C for 30 min, 42 °C for 30 min, 85 °C for 5 min. The miRNA expression levels were analyzed by TaqMan MicroRNA probes (ABI, USA) using MMX(2x) of FastStart Essential DNA Probes Master (Roche, Basel, Switzerland) on a LightCycler 98 system (Roche) with the following reaction conditions: enzyme activation at 95 °C for 600 s, followed by 60 cycles of two-step amplification at 95 °C for 10 s and 60 °C for 60 s. All reactions were performed in triplicate. RNU6B was used as a control for miRNA.

### Protein extraction and western blot analysis

MCF7 cells were harvested before adding radioimmunoprecipitation assay (RIPA) buffer. Samples were mixed with Laemmli sample buffer (Bio-rad, Hercules, CA, USA), heated at 95 °C for 5 min, separated by 10% sodium dodecyl sulfate-polyacrylamide gel electrophoresis (SDS-PAGE), and transferred to polyvinylidene fluoride (PVDF) membranes (Millipore, Billerica, MA, USA). Membranes were incubated overnight at 4 °C with rabbit polyclonal antibodies to TGFβRII (1:100; Santa Cruz Biotechnology, Dallas, TX, USA). After washing with 0.5X tris-buffered saline with tween 20 (TBST) for 1 h, blots were incubated for 1 h at room temperature with horseradish peroxidase-conjugated anti-rabbit secondary antibody (1:3000; Santa Cruz Biotechnology), and protein bands were visualized using an Pierce™ BCA Protein Assay Kit (Thermo Fisher Scientific). A β-actin antibody (1:1000; Cell Signaling Technology, Danvers, MA, USA) was used to confirm comparable loading. The density of each protein band was determined using Fujifilm Multi Gauge version 3.0 software.

### Statistical analysis

Baseline patient group was compared using Mann Whitney *U* test and Fisher’s exact test. MiRNA data were presented as mean ± SD. Student’s *t* test and one-way ANOVA analyses were performed to analyze the differences between the groups. Correlation between miRNA and target gene was done by Spearman correlation analysis. Statistical analyses were performed using SPSS 24.0 software (IBM Corp., Armonk, NY).

## Results

### Patient characteristics

Forty tissue samples were collected, including 10 patients each diagnosed with non-recurrent DCIS (group A), recurrent DCIS (group B), pure DCIS (group C), and synchronous DCIS with adjacent IDC (group D). The baseline characteristics of patients are summarized in Table 1. Group B patients were significantly younger compared to group A (*P* = 0.009). In addition, group A patients showed relatively lower Ki-67 index level (*P* = 0.043), lower nuclear grade in malignant cells with a predominance for the luminal A subtype compared to group B. Group C and D patients were similar in age distribution, with no differences among subtype classification. Group C patients showed lower Ki-67 index level compared to group D, but without statistical significance.

**Table 1:** Baseline patient characteristics

	Primary DCIS (group A)	Recurrent DCIS (group B)	P value	Pure DCIS (group C)	DCIS adjacent to IDC (group D)	*P* value
No. of patients	10	10		10	10	
Age (years)			0.009			0.73
Median	49.5	43		48.5	47.5	
Range	41–55	30–70		40–71	37–75	
Size (longest diameter)			0.393			0.32
Median	2	1.5		2.7	2.05	
Range	1–3.8	0.9–3.7		1.4–5.6	0.11–7.0	
Nuclear grade			0.17			0.99
1	6	2		2	3	
2	1	3		4	2	
3	3	4		4	5	
Margin						
Negative	10	9		7	9	
Close	0	0		3	1	
Unknown	0	1		0	0	
Subtype			0.321			0.99
Luminal A	8	4		4	5	
Luminal B (HER2 negative)	0	1		0	0	
Luminal B (HER2 positive)	2	1		2	4	
HER2 positive	0	1		3	1	
Basal-like	0	1		1	0	
Subtype according to hormonal status						
ER positive	9	7		6	9	
PR positive	10	6		6	7	
HER2 positive	2	2		4	5	
Ki67			0.04			0.36
Low (≤ 20%)	9	4		9	6	
High (> 20%)	1	4		1	4	
Unknown	0	2		0	0	

*ER* estrogen receptor, *PR* progesterone receptor, *HER2* human epithelial growth factor 2

### miRNA microarray analysis

MiRNA microarrays were performed to analyze the global miRNA expression profiles for all 40 samples (groups A–D). Among 2,578 mature human miRNAs represented on the microarray, 665 (25.8%) of the identified miRNAs were expressed at least once.

**Fig. 1:**
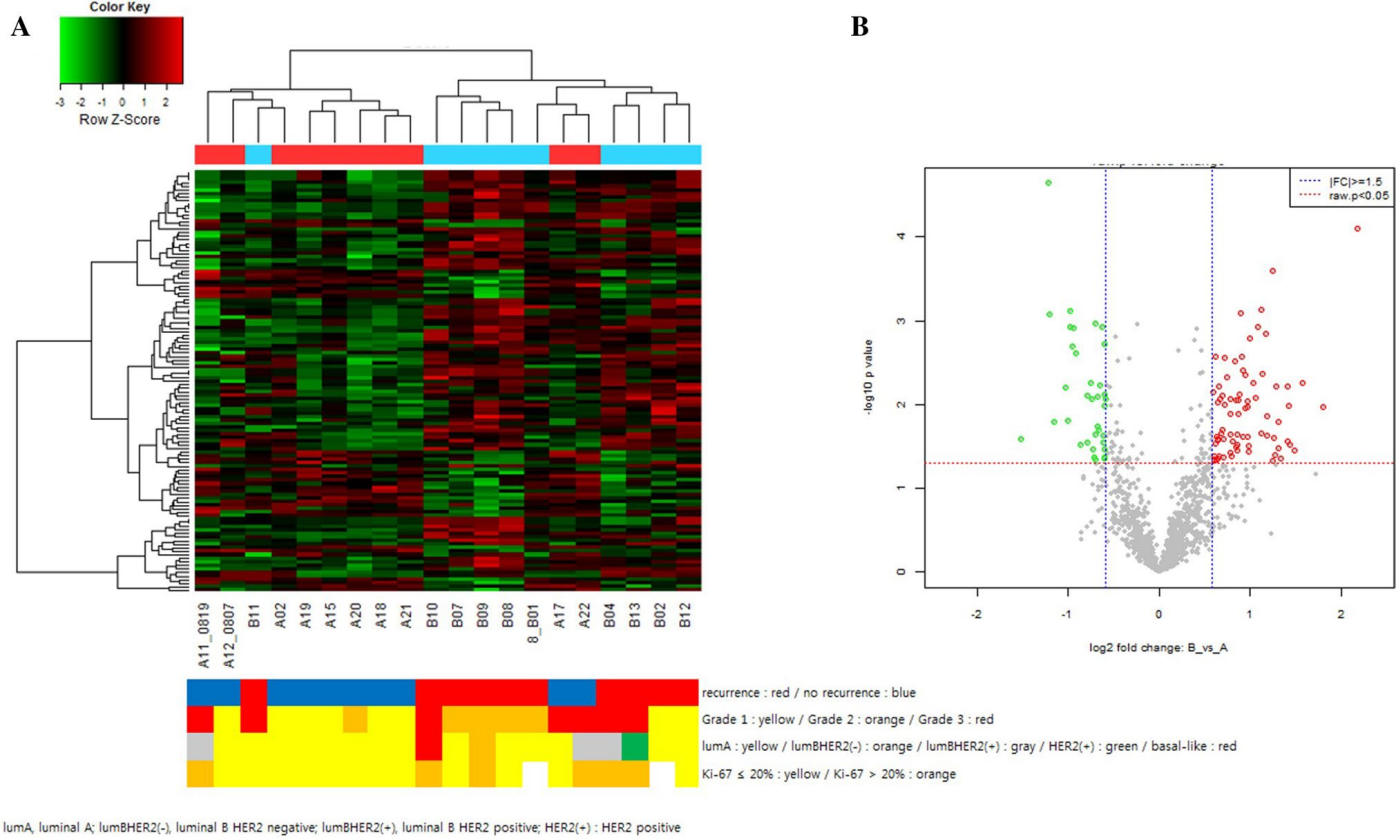
Scaled-down representation of the 2578 mature human miRNA, presented by hierarchical clustering (Euclidean Method, Complete Linkage) (**a**) and Volcano plot of miRNA expression level (**b**) between groups A and B

To identify recurrence-associated miRNA changes, we compared the expression levels of the miRNAs in the patients with non-recurrent (group A) and recurrent (group B) DCIS. Unsupervised hierarchical clustering analysis showed clearly different miRNA expression patterns between the two groups, which were divided into two clusters, except for three cases (Fig. 1a). The expression changes of individual miRNAs are shown as a volcano plot in Fig. 1b. Overall, 27 miRNAs were up-regulated in group B with an absolute fold change (|*fc*|) ≥ 1.5 and 26 miRNAs were down-regulated with |*fc*|≤ 1.5, indicating significant differences (raw *P* values < 0.05; Supplementary Table 1).

**Fig. 2:**
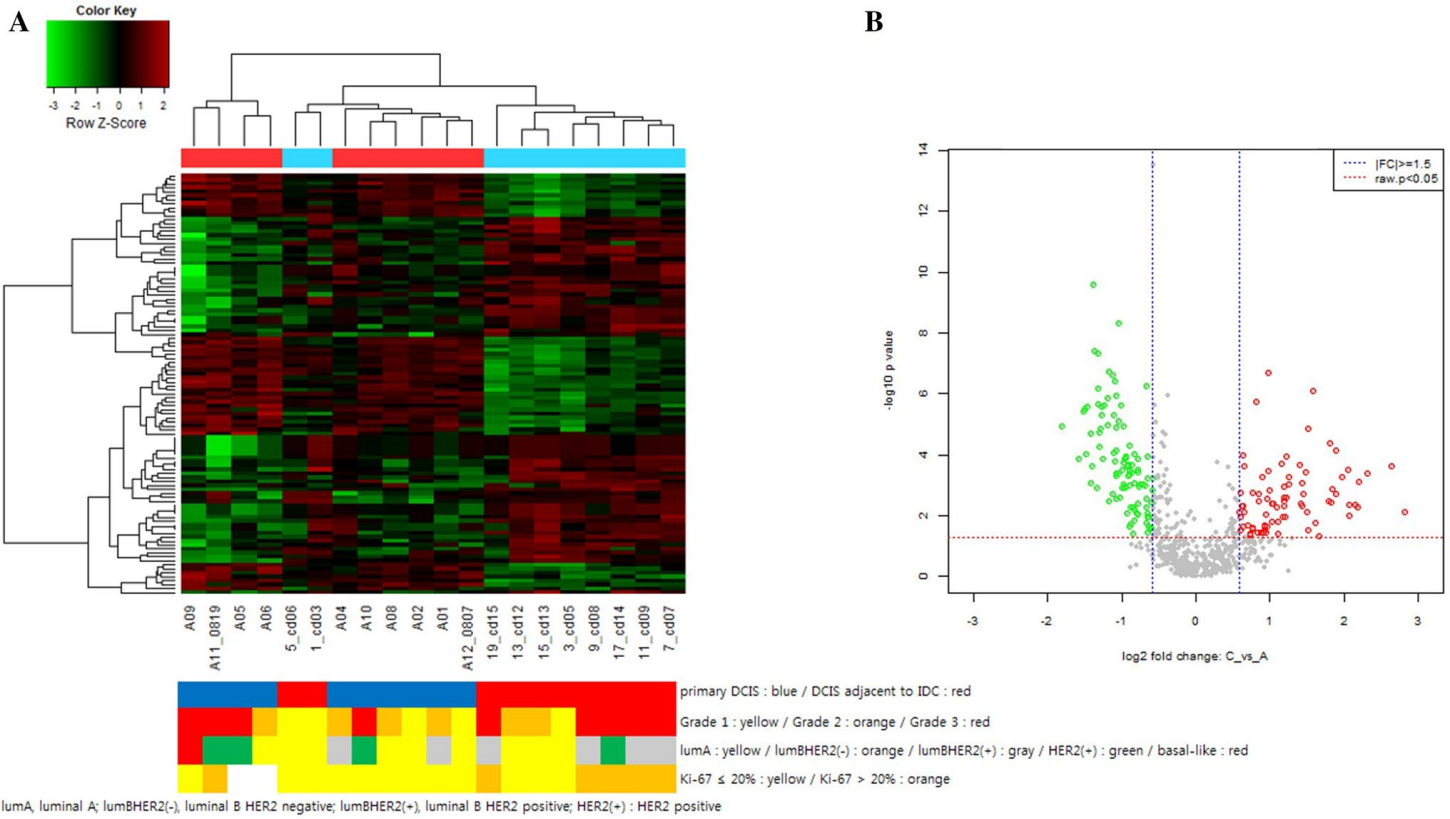
Scaled-down representation of the 2578 mature human miRNA, presented by hierarchical clustering (Euclidean Method, Complete Linkage) (**a**) and volcano plot of miRNA expression level (**b**) between groups C and D

To identify progression-associated miRNA changes, we further analyzed the expression levels of miRNAs in patients diagnosed with pure DCIS (group C) and synchronous DCIS with adjacent IDC (group D). Unsupervised hierarchical clustering analysis clearly showed different miRNA expression patterns between the groups, which were divided into two clusters with exception for two cases (Fig. 2a). The expression changes of individual miRNAs are plotted as a volcano plot in Fig. 2b. Overall, 32 miRNAs were up-regulated in group C with a |fc| ≥ 1.5 and 42 miRNAs were down-regulated with a |fc| ≤ 1.5 (Supplementary Table 2).

Of these significantly dysregulated miRNAs detected between group B and group D, 17 miRNAs were included in both groups and showed similar alterations: seven miRNAs (miR-17-5p, -20a-5p, -103a-3p, -106a-5p, -107, -106b-5p, and -7641) were up-regulated and 10 miRNAs (miR-4281, -4534, -4689, -6124, -6127, -6165, -6776-5p, -6870-5p, -6879-5p, and -6891-5p) were down-regulated (Table 2), suggesting the possibility in the recurrence and/or progression of DCIS. Among these 17 miRNAs, we further focused on the miR-106b and miR-17 clusters. We hypothesized these two clusters might have a synergistic or cooperative effect in DCIS recurrence and/or progression by sharing a common seed sequence (AAGUGCU). MiR-106b-5p and miR-17-5p were selected to represent miR-106b-25 cluster and miR-17-92 cluster. MiR-20a-5p also belonged to miR-17-92 cluster, but we selected miR-17-5p for further analysis considering literature reporting up-regulation of miR-17-5p in breast cancer, and association to cancer cell proliferation [19, 20].

**Table 2:** Dysregulated miRNAs in recurrent DCIS and synchronous DCIS with IDC

Transcript ID (array design)	B/A fold change	D/C fold change	Sequence
hsa-miR-106b-5p	2.37952	4.99945	U*AAAGUGCU*GACAGUGCAGAU
hsa-miR-17-5p	1.74321	2.6783	C*AAAGUGCU*UACAGUGCAGGUAG
hsa-miR-20a-5p	1.78973	3.5777	U*AAAGUGCU*UAUAGUGCAGGUAG
hsa-miR-103a-3p	1.63249	2.06554	AGCAGCAUUGUACAGGGCUAUGA
hsa-miR-106a-5p	1.89352	2.69959	AAAAGUGCUUACAGUGCAGGUAG
hsa-miR-107	1.71775	2.3866	AGCAGCAUUGUACAGGGCUAUCA
hsa-miR-7641	1.95981	2.26739	UUGAUCUCGGAAGCUAAGC
hsa-miR-4281	− 1.5725	− 2.1403	GGGUCCCGGGGAGGGGGG
hsa-miR-4534	− 2.0479	− 2.5305	GGAUGGAGGAGGGGUCU
hsa-miR-4689	− 1.6231	− 2.9964	UUGAGGAGACAUGGUGGGGGCC
hsa-miR-6124	− 1.5242	− 2.2863	GGGAAAAGGAAGGGGGAGGA
hsa-miR-6127	− 1.5913	− 2.8221	UGAGGGAGUGGGUGGGAGG
hsa-miR-6165	− 1.9717	− 2.6704	CAGCAGGAGGUGAGGGGAG
hsa-miR-6766-5p	− 1.6595	− 2.8509	CGGGUGGGAGCAGAUCUUAUUGAG
hsa-miR-6870-5p	− 1.9992	− 2.6661	UGGGGGAGAUGGGGGUUGA
hsa-miR-6879-5p	− 1.5138	− 2.105	CAGGGCAGGGAAGGUGGGAGAG
hsa-miR-6891-5p	− 1.5146	− 2.4686	UAAGGAGGGGGAUGAGGGG

### miRNA target prediction and nCounter PanCancer pathway analysis

For the genetic target prediction of miR-106b-5p and miR-17-5p, pre-formed miRNA databases were searched for possible direct targets shared by miR-106b-5p and miR-17-5p. Several target genes were predicted, including *TGFβR2, SMAD4*, and *RBL2* (Supplementary Table 3). Furthermore, *SMAD7* was included as one of a target of miR-106b-5p and miR-17-5p, based on reports which *SMAD7* might influence cancer cell proliferation and metastasis in hepatocellular carcinoma [21].

**Fig. 3:**
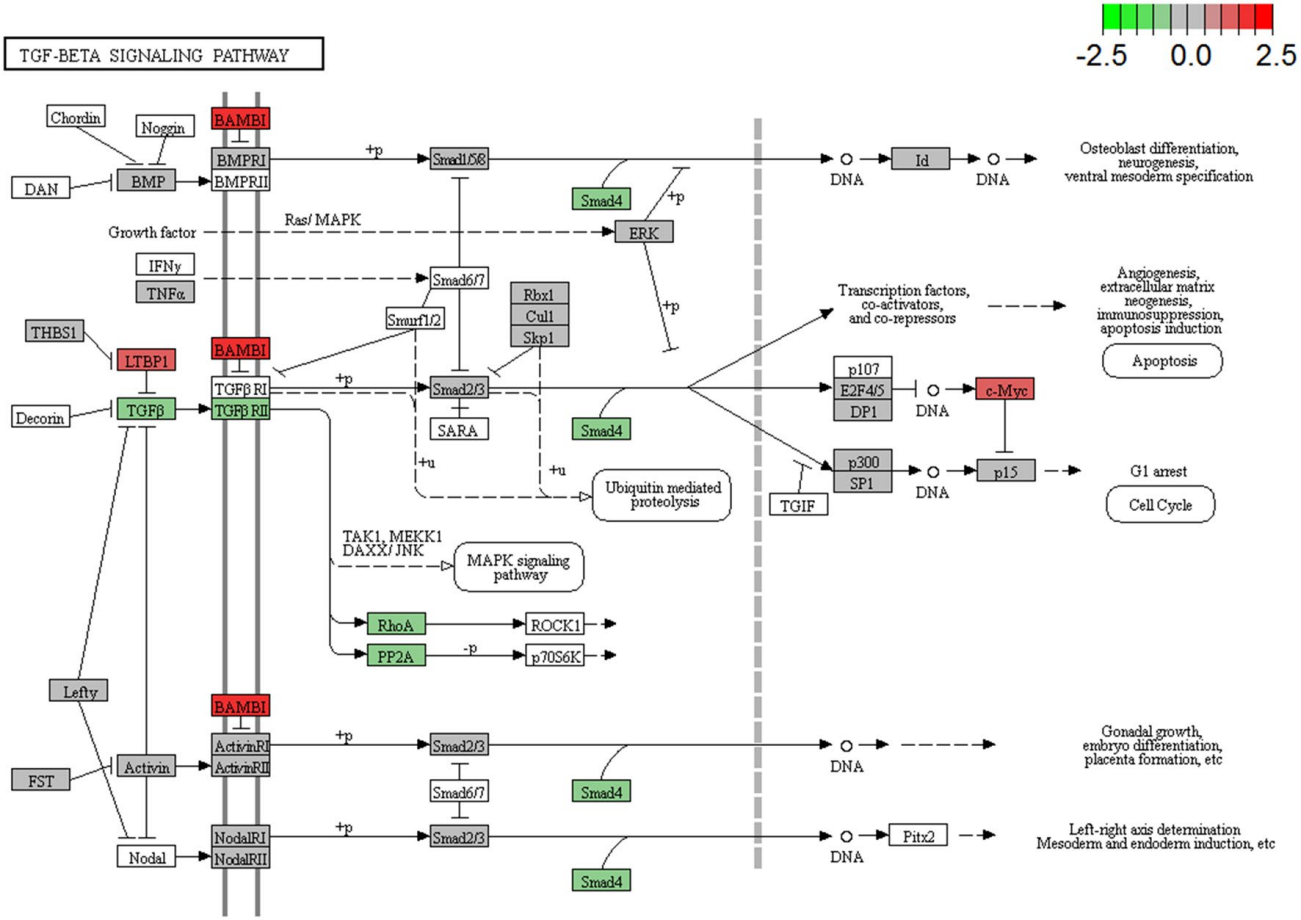
Results of Pancancer Pathway Panel analysis. In TGF-β signaling pathway, group B showed downregulation of TGF-β and TGFβRII, which is depicted in green color. Other genes involved in TGF-β signaling pathways were also down-regulated

We next evaluated the expression patterns of the cancer-associated genes between recurrent and non-recurrent tissue samples using PanCancer pathway analysis (Nanostring Technologies, Inc.). During analysis, false discovery rate was controlled by adjusting the *P* value using the Benjamini–Hochberg algorithm. We focused on TGF-β pathway expression changes in recurrent DCIS tissues compared to the non-recurrent DCIS tissues. Gene expression levels of *TGFβRII, TGFβ,* and *SMAD4* were lower in recurrent DCIS tissues compared to non-recurrent tissues in TGF-β pathway (Fig. 3). Among prior mentioned genes, *TGFβRII* and *SMAD4* were significantly down-regulated. Based on PanCancer pathway analysis, we hypothesized that the TGF-β pathway might contribute to malignant transformation in breast tissue.

Correlation between miRNA and target gene was analyzed. MiR-17-5p showed negative correlation with *SMAD4* (*r* = − 0.519, *P* = 0.027, Spearman’s correlation), and tendency for negative correlation with *TGFβRII* (*r* = − 0.205, *P* = 0.414, Spearman’s correlation). MiR-106b-5p also showed negative correlation with *TGFβRII* and *SMAD4* (*TGFβRII r* = − 0.593, *P* = 0.009; *SMAD4 r* = − 0.546, *P* = 0.019, Spearman’s correlation).

Integrating the common findings in target prediction database and PanCancer pathway analyses, we selected *TGFβRII* as a main potential direct target of both miR-106b-5p and miR-17-5p, and hypothesized that aberrant expression of these miRNAs might down-regulate the TGF-β pathway to play an important role in the recurrence and progression of DCIS. To evaluate this hypothesis, we transfected these miRNAs into a breast cancer cell line and determined their effects on the expression of proteins involved in this pathway.

### Transfection confirmation with reverse transcription quantitative polymerase chain reaction (RT-qPCR)

**Fig. 4:**
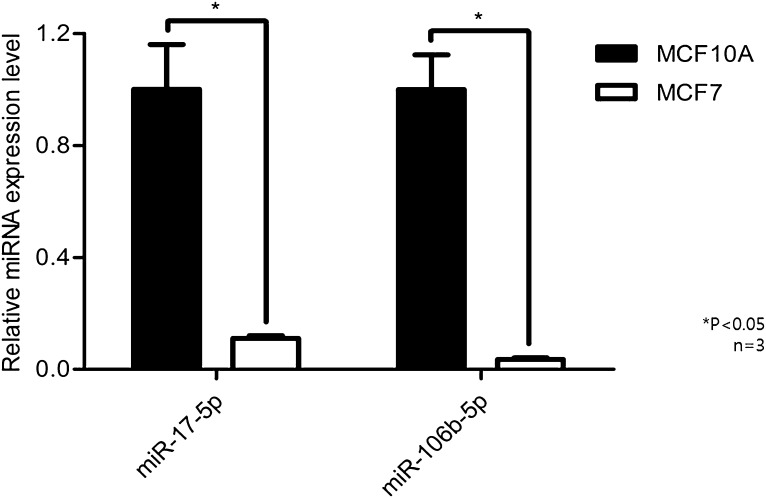
Expression level of miR-17-5p or miR-106b-5p in MCF10A and MCF7. Baseline level of miR-17-5p or miR-106-5p. MCF10A or MCF7 cells were harvested and levels of each miRNA were measured by qRT-PCR. Relative gene expression was calculated according to the comparative Ct method, using RNU6B as an internal control (*n* = 3). Error bars indicate standard deviation (SD)

**Fig. 5:**
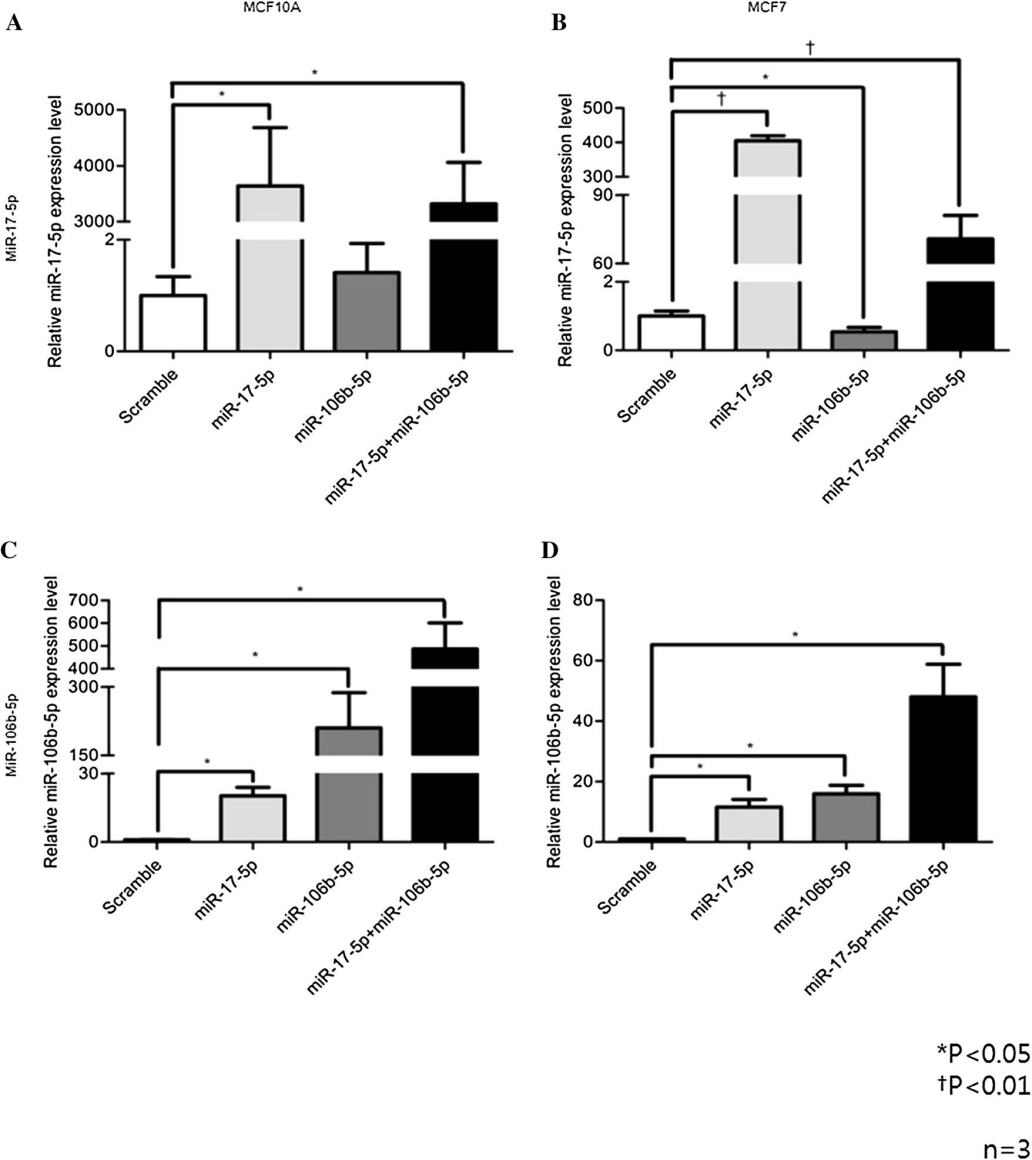
Expression of miR-17-5p or miR-106b-5p after each miRNA mimic treatment. **a**–**d** Up-regulation of miR-17-5p or miR-106-5p levels after each miRNA mimic treatment. MCF10A or MCF7 cells were transfected with 20 nM miR-17-5p, miR-106b mimics, or the scrambled control, and levels of each miRNA were measured by qRT-PCR. Relative gene expression was calculated according to the comparative Ct method, using RNU6B as an internal control (*n* = 3). Error bars indicate standard deviation (SD)

The MCF-7 cell line was selected for analysis, which is a breast cancer cell line that has relatively low-invasive potential; MCF-10A cells were selected as a negative control demonstrating normal breast tissue. The baseline levels of miR-106b and miR-17 expression for the two cell lines are shown in Fig. 4. MCF-7 and MCF-10A cells were each transfected with miR-106b and miR-17 mimics each, mixture of miR-106b and miR-17 mimics, and scrambled control of miRNA as negative inhibitor. After transfection with either mimic, both cell lines showed increased expression of miR-17 or miR-106b. When transfected with mixture of miR-106b and miR-17, there were no difference of miR-17 expression compared to sole miR-17 transfection in both cell lines. However, miR-106b expression was increased in both cell lines when transfected with mixture of both miRNAs compared to single miR-106b-transfected cell lines (Fig. 5).

### Expression of proteins related to the TGF-β pathway

**Fig. 6:**
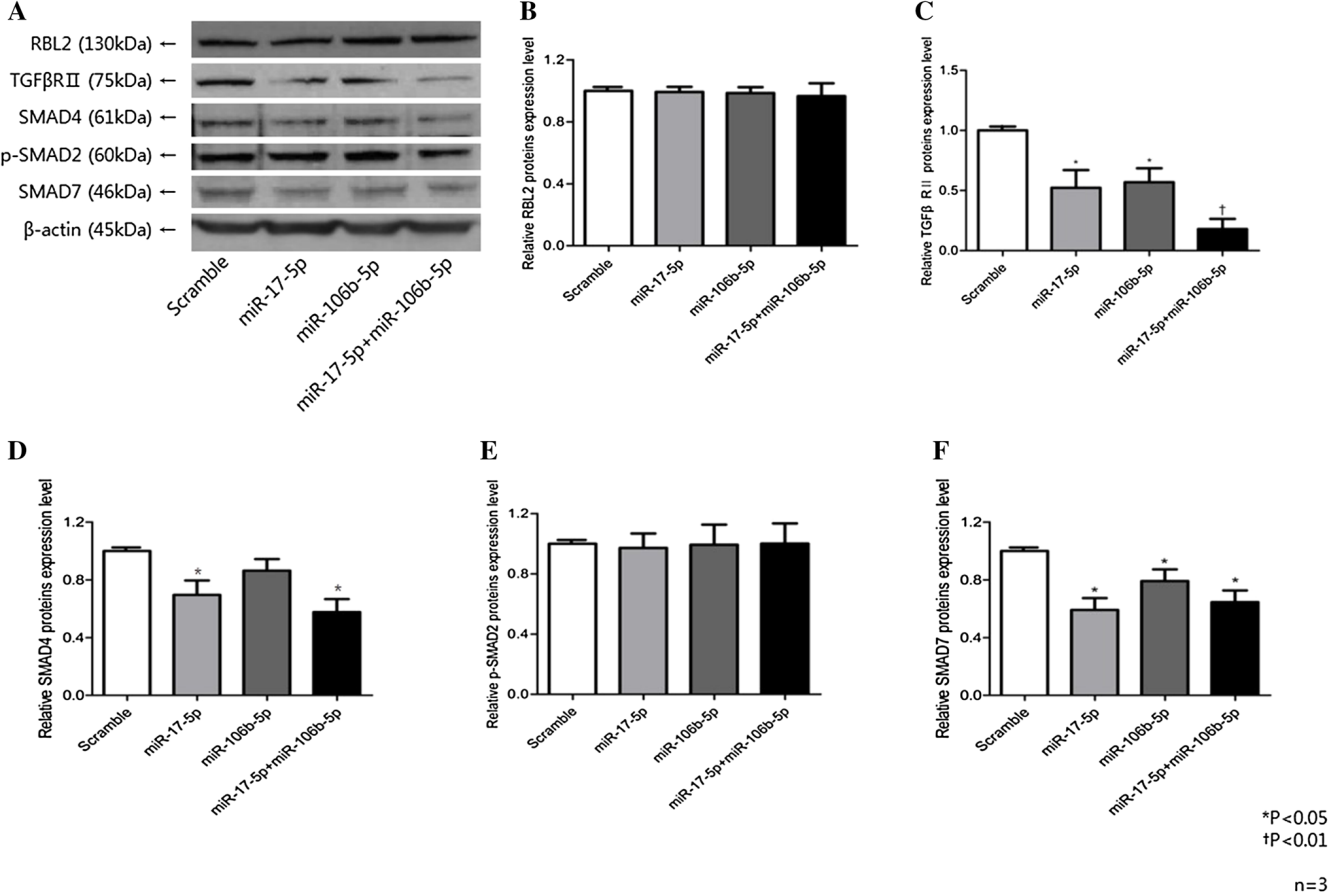
Effects of miR-17-5p and miR-106b-5p mimics on expression of TGFβRII protein. MCF7 cells were transfected with 20 nM miR-17-5p, miR-106b-5p mimics, or the scrambled control. Cell lysates were examined by Western blot analysis with an anti-TGFβRII polyclonal antibody (1:100) at 48 h post-transfection; an anti-β-actin antibody (1:1000) was used to normalize protein loading. Three sets of independent experiments were performed, and representative results are shown. The density of each protein band was quantified using Fujifilm Multi Gauge software version 3.0 and expressed as a ratio to the density of the band from the scrambled control. Error bars indicate standard deviation (SD)

We examined whether miR-106b and miR-17 regulated the expression of *TGFβRII* to exert a suppressive effect on the TGF-β pathway in MCF-7 cell line through western blot analysis after transfection of the miRNA mimics. MCF-7 cells transfected with miR-106b and miR-17 mimics showed decreased expression levels of *TGFβRII* compared to control (Fig. 6), in accordance with the results of the Nanostring PanCancer pathway analysis. When transfected with mixture of miR-106b and miR-17 mimics, *TGFβRII* showed further suppression compared to single miRNA mimic transfected state. *SMAD4* showed relative suppression when transfected with both miRNA mimics, and *SMAD7* was suppressed when transfected with single miR-17-5p mimic.

## Discussion

Although DCIS is well-known precancerous lesion that could eventually progress to IDC, the underlying mechanism of malignant transformation remains largely unknown. This gap in knowledge is largely due to rare incidence of recurrent DCIS or IDC after effective primary treatment. In this study, we identified miR-106b-5p and miR-17-5p as potential factors contributing to the recurrence and progression of DCIS by targeting and down-regulating the TGF-β pathway in breast cancer. MiR-17-5p is located to miR-17-92 cluster, and miR-106b belongs to miR-106b-25 cluster. MiR-17-92 cluster is known as an oncogenic miRNA cluster first identified [22]. MiR-106b-25 is a paralog of miR-17-92 cluster and shares same oncogenic role in many mammalian cells [23]. Prior miRNA clusters are both related to TGF-β pathway and alteration of TGF- β also influence miR-17-92 and miR-106b-25 clusters [24]. Although miR-17 and miR-106b-5p are located at different miRNA clusters and different chromosomes, they are classified as miR-17 family based on same seed sequence. By sharing the same seed sequence, miR-17 and miR-106b-5p may target similar genes and consequently influence common pathway during oncogenesis [25]. Therefore, we assumed two miRNAs may have similar or synergistic effect in TGF-β pathway.

Haakensen et al. [12] also found that miR-106-5p was up-regulated in DCIS and IDC tissues compared to normal tissue samples, suggesting this miRNA might act as an oncomir during DCIS and IDC development. MiR-17-5p has been reported to be associated with breast cancer cell proliferation, migration, and invasion, but its role as an oncomir or tumor suppressor was unknown with conflicting results [26–28]. Enlery et al. have reported miR-17-92 cluster is associated with breast cancer proliferation [29]. Other than miR-106-5p and miR-17-5p, miRNAs such as miR-375, miR-592, and miR-135a also influence breast cancer proliferation by modulating *ESR1, ERBB4* pathway [30], and miR-135a function as prognostic marker in ER-positive breast cancer [31]. Among previous miRNAs, miR-17-5p and miR-106-5p share common seed sequence and this may explain their similar role in breast cancer proliferation. In our study, both miR-106-5p and miR-17-5p were up-regulated in recurrent DCIS and synchronous DCIS adjacent to IDC tissues compared to primary DCIS. Together, these findings suggest that two miRNAs may play a role in very early breast carcinogenesis, extending to early cancer recurrence, progression, and proliferation of breast cancer thereafter.

In our study, TGF-β pathway was dysregulated in recurrent DCIS tissues compared to primary DCIS tissues in PanCancer Pathway panel results and the proliferation potential was significantly enhanced in recurrent tissues based on Ki-67 immunohistochemical stain. MiRNA target predicting database supported our findings, suggesting *TGFβRII* as a common target of miR-106b-5p and miR-17-5p. Thus, our results are in accordance with previous studies demonstrating that miR-106b-5p and miR-17-5p function as oncomirs in the formation and development of cancer including breast cancer, with an indicated association with the TGF-β pathway [15, 21, 26, 32, 33]. These findings led us to hypothesize that miR-106b-5p and miR-17-5p may have a role in breast cancer carcinogenesis, especially from a very early stage, via TGF-β pathway regulation to influence cancer cell proliferation activity.

*TGFβRII* is one of the main receptors of the TGF-β pathway [34]. Based on decreased expression of *TGFβRII* and down-regulated TGF-β pathway genes in PanCancer pathway panel analysis, miR-106b-5p and miR-17-5p appeared to regulate the TGF-β pathway and *TGFβRII* as likely as their common direct target. This hypothesis was verified by in vitro study given that miRNA-transfected MCF-7 breast cancer cells showed decreased expression of the TGF-β-related proteins such as TGFβRII, SMAD4, and SMAD7 compared to control, in concordance with previous literature [24].

Contrary to our study, there is conflicting result about the role of TGF-β pathway in breast cancer progression. Chen et al. showed overexpression of miR-21 in MCF7 and Hs578T breast cancer cell line might be associated with activation of TGF-β pathway by suppressing SMAD7 during tumor progression [11]. This conflicting result may be due to the complex nature of TGF-β pathway. As previously mentioned, TGF-β has paradoxical role according to surrounding cellular environment and the status of cancer progression [14, 15]. Furthermore, there are heterogeneous miRNAs influencing progression of DCIS to invasive cancer [35], and extracellular matrix consisted of fibroblast and immune cells also affect recurrence and progression [36]. MiRNAs target TGF-β pathway at different environment and progression stages, and these various situations may lead to paradoxical mechanism of TGF-β pathway.

There are some limitations of our study. As mentioned above, because of the rarity of recurrent DCIS tissue, the sample size of total patient tissues is relatively small. We could only collect 10 recurrent DCIS tissues in 2 major tertiary institutions during 10 years of follow-up period for comparison with other types of DCIS. This small sample size requires careful interpretation of the study results. Considering the rare incidence of recurrent DCIS during long-term follow-up, our analysis can act as a pilot study for initiating large multicenter study for follow-up analysis. Among DCIS tissues, although most of the samples were luminal A or B type, there were few HER2-positive or triple-negative cases among recurrent DCIS and synchronous DCIS samples. We used low-invasive MCF-7 representing hormone receptor-positive DCIS, because there were no established conventional DCIS cell lines available. Other than sample size, this different subtype also should be considered during interpretation. For in-depth study of the role of miR-106b-5p and miR-17-5p in evolution and recurrence of DCIS in human, in vitro study using patient-derived recurrent DCIS cell line and in vivo study using patient-derived xenograft (PDX) model may be needed. We are planning on multicenter study for collection of recurrent DCIS patient sample and constructing patient-driven recurrent DCIS cell line and PDX model for in-depth study.

In summary, we verified the potential role of miR-106b-5p and miR-17-5p in the recurrence or progression of DCIS via down-regulating the TGF-β pathway. Validation of these miRNAs in vitro suggested TGFβRII as the direct target, in line with previous reports. Considering the scarcity of recurrent DCIS tissues, the direct validation of this dysregulation in miRNA expression patterns from the recurrent DCIS or DCIS adjacent to synchronous IDC tissue as well as the oncogene panel analysis has particular value. Based on these results, miR-106b-5p and miR-17-5p could serve as useful biomarkers for selecting patients who are more prone to recurrence or progression of DCIS. Detecting miR-106b-5p or miR-17-5p on surgical specimens during diagnosis, more detailed surveillance with tailored follow-up of taking hormonal agent with better compliance can be offered to patients, and this may lead to reduced incidence of DCIS or invasive cancer during follow-up.

## Electronic supplementary material

Below is the link to the electronic supplementary material.

Supplementary material 1 (DOCX 13 KB)

Supplementary material 2 (DOC 67 KB)

Supplementary material 3 (DOC 30 KB)
