# Supplementary material for: miR-106b-5p and miR-17-5p could predict recurrence and progression in breast ductal carcinoma in situ based on the transforming growth factor-beta pathway
Source: Breast Cancer Res Treat. 2019 Apr 15;176(1):119–30. doi: 10.1007/s10549-019-05192-1 (PMC6548759; doi:10.1007/s10549-019-05192-1)
Supplement: Supplementary file 1 — Supplementary material 1 (DOCX 13 KB) [file 10549_2019_5192_MOESM1_ESM.docx]

Supplementary Table 1. Dysregulated miRNAs in non-recurrent DCIS and recurrent DCIS

| No. | Transcript ID (Array Design) | B/A fold change | No. | Transcript ID (Array Design) | B/A fold change |
| --- | --- | --- | --- | --- | --- |
| 1 | hsa-let-7f-1-3p | 1.81987 | 1 | hsa-miR-375 | -2.8608 |
| 2 | hsa-miR-17-5p | 1.74321 | 2 | hsa-miR-328-5p | -1.5045 |
| 3 | hsa-miR-20a-5p | 1.78973 | 3 | hsa-miR-4271 | -2.2201 |
| 4 | hsa-miR-25-3p | 2.40514 | 4 | hsa-miR-4281 | -1.5725 |
| 5 | hsa-miR-31-5p | 2.65575 | 5 | hsa-miR-3911 | -1.6888 |
| 6 | hsa-miR-93-5p | 1.97896 | 6 | hsa-miR-4534 | -2.0479 |
| 7 | hsa-miR-100-5p | 2.47408 | 7 | hsa-miR-4656 | -1.5202 |
| 8 | hsa-miR-103a-3p | 1.63249 | 8 | hsa-miR-4669 | -1.6743 |
| 9 | hsa-miR-106a-5p | 1.89352 | 9 | hsa-miR-4689 | -1.6231 |
| 10 | hsa-miR-107 | 1.71775 | 10 | hsa-miR-4788 | -2.334 |
| 11 | hsa-miR-199a-3p | 4.51297 | 11 | hsa-miR-5195-3p | -1.6446 |
| 12 | hsa-miR-181a-5p | 2.48482 | 12 | hsa-miR-5100 | -1.8203 |
| 13 | hsa-miR-181b-5p | 2.52197 | 13 | hsa-miR-5739 | -1.5198 |
| 14 | hsa-miR-199b-3p | 4.51297 | 14 | hsa-miR-6085 | -2.308 |
| 15 | hsa-miR-214-3p | 2.16819 | 15 | hsa-miR-6124 | -1.5242 |
| 16 | hsa-miR-221-3p | 2.66597 | 16 | hsa-miR-6127 | -1.5913 |
| 17 | hsa-miR-106b-5p | 2.37952 | 17 | hsa-miR-6165 | -1.9417 |
| 18 | hsa-miR-106b-3p | 3.48499 | 18 | hsa-miR-6766-5p | -1.6595 |
| 19 | hsa-miR-151a-3p | 2.70403 | 19 | hsa-miR-6785-5p | -1.6037 |
| 20 | hsa-miR-1825 | 2.79105 | 20 | hsa-miR-6829-5p | -1.7281 |
| 21 | hsa-miR-1910-5p | 2.39605 | 21 | hsa-miR-6870-5p | -1.9992 |
| 22 | hsa-miR-3175 | 1.73445 | 22 | hsa-miR-6879-5p | -1.5138 |
| 23 | hsa-miR-3620-5p | 1.58804 | 23 | hsa-miR-6891-5p | -1.5146 |
| 24 | hsa-miR-3651 | 1.56058 | 24 | hsa-miR-7106-5p | -1.9216 |
| 25 | hsa-miR-4507 | 1.83102 | 25 | hsa-miR-7150 | -1.6273 |
| 26 | hsa-miR-6750-5p | 1.56819 | 26 | hsa-miR-8072 | -1.5158 |
| 27 | hsa-miR-7641 | 1.95981 |  |  |  |
