# Supplementary material for: miR-106b-5p and miR-17-5p could predict recurrence and progression in breast ductal carcinoma in situ based on the transforming growth factor-beta pathway
Source: Breast Cancer Res Treat. 2019 Apr 15;176(1):119–30. doi: 10.1007/s10549-019-05192-1 (PMC6548759; doi:10.1007/s10549-019-05192-1)
Supplement: Supplementary file 2 — Supplementary material 2 (DOC 67 KB) [file 10549_2019_5192_MOESM2_ESM.doc]

Supplementary Table 2. Dysregulated miRNAs in pure DCIS and synchronous DCIS with adjacent IDC

| No | Transcript ID (Array Design) | D/C fold change | No. | Transcript ID (Array Design) | D/C fold change |
| --- | --- | --- | --- | --- | --- |
| 1 | hsa-mir-16-5p | 2.98999 | 1 | hsa-miR-197-5p | -2.3916 |
| 2 | hsa-miR-17-5p | 2.6783 | 2 | hsa-miR-214-3p | -2.6001 |
| 3 | hsa-miR-20a-5p | 3.57977 | 3 | hsa-miR-1224-5p | -2.2525 |
| 4 | hsa-miR-22-3p | 2.87583 | 4 | hsa-miR-1229-5p | -2.0347 |
| 5 | hsa-miR-26a-5p | 2.38551 | 5 | hsa-miR-1268a | -2.1684 |
| 6 | hsa-miR-27a-3p | 3.52355 | 6 | hsa-miR-3162-5p | -2.0973 |
| 7 | hsa-miR-29a-3p | 4.20081 | 7 | hsa-miR-4270 | -2.1135 |
| 8 | hsa-miR-30a-5p | 7.04891 | 8 | hsa-miR-4281 | -2.1403 |
| 9 | hsa-miR-103a-3p | 2.06554 | 9 | hsa-miR-1268b | -2.097 |
| 10 | hsa-miR-106a-5p | 2.69959 | 10 | hsa-miR-4443 | -2.3984 |
| 11 | hsa-miR-107 | 2.3866 | 11 | hsa-miR-4459 | -2.0115 |
| 12 | hsa-miR-30c-5p | 4.197 | 12 | hsa-miR-4487 | -2.0552 |
| 13 | hsa-miR-30d-5p | 2.12499 | 13 | hsa-miR-2392 | -2.7715 |
| 14 | hsa-miR-34a-5p | 2.05129 | 14 | hsa-miR-4534 | -2.5305 |
| 15 | hsa-miR-210-3p | 2.21313 | 15 | hsa-miR-1587 | -2.4884 |
| 16 | hsa-miR-15b-5p | 3.71498 | 16 | hsa-miR-4651 | -2.1308 |
| 17 | hsa-miR-30b-3p | 6.22408 | 17 | hsa-miR-4689 | -2.9964 |
| 18 | hsa-miR-141-3p | 4.17737 | 18 | hsa-miR-5196-5p | -2.4327 |
| 19 | hsa-miR-191-5p | 2.04981 | 19 | hsa-miR-5739 | -2.801 |
| 20 | hsa-miR-125a-5p | 2.34207 | 20 | hsa-miR-6124 | -2.2863 |
| 21 | hsa-miR-195-5p | 4.55858 | 21 | hsa-miR-6127 | -2.8221 |
| 22 | hsa-miR-106b-5p | 4.99945 | 22 | hsa-miR-6132 | -2.0985 |
| 23 | hsa-miR-99b-5p | 2.28811 | 23 | hsa-miR-6165 | -2.6704 |
| 24 | hsa-miR-342-3p | 4.47271 | 24 | hsa-miR-6749-5p | -2.4108 |
| 25 | hsa-miR-193b-3p | 2.21082 | 25 | hsa-miR-6752-5p | -2.2871 |
| 26 | hsa-miR-92b-3p | 2.78347 | 26 | hsa-miR-6756-5p | -2.4956 |
| 27 | hsa-miR-574-3p | 2.69098 | 27 | hsa-miR-6763-5p | -2.3669 |
| 28 | hsa-miR-151b | 3.47539 | 28 | hsa-miR-6776-5p | -2.8509 |
| 29 | hsa-miR-3128 | 4.59644 | 29 | hsa-miR-6779-5p | -2.0607 |
| 30 | hsa-miR-3201 | 2.85084 | 30 | hsa-miR-6782-5p | -2.4833 |
| 31 | hsa-miR-7641 | 2.26739 | 31 | hsa-miR-6794-5p | -2.1437 |
| 32 | hsa-miR-8084 | 3.16625 | 32 | hsa-miR-6798-5p | -2.2544 |
|  |  |  | 33 | hsa-miR-6802-5p | -2.4779 |
|  |  |  | 34 | hsa-miR-6812-5p | -2.1643 |
|  |  |  | 35 | hsa-miR-6813-5p | -2.6484 |
|  |  |  | 36 | hsa-miR-6769b-5p | -2.5843 |
|  |  |  | 37 | hsa-miR-6870-5p | -2.6661 |
|  |  |  | 38 | hsa-miR-6879-5p | -2.105 |
|  |  |  | 39 | hsa-miR-6891-5p | -2.4686 |
|  |  |  | 40 | hsa-miR-6893-5p | -3.5028 |
|  |  |  | 41 | hsa-miR-4433b-3p | -2.0619 |
|  |  |  | 42 | hsa-miR-7845-5p | -2.0947 |
