# Supplementary material for: miR-106b-5p and miR-17-5p could predict recurrence and progression in breast ductal carcinoma in situ based on the transforming growth factor-beta pathway
Source: Breast Cancer Res Treat. 2019 Apr 15;176(1):119–30. doi: 10.1007/s10549-019-05192-1 (PMC6548759; doi:10.1007/s10549-019-05192-1)
Supplement: Supplementary file 3 — Supplementary material 3 (DOC 30 KB) [file 10549_2019_5192_MOESM3_ESM.doc]

Supplementary Table 3. Target genes predicted based on miRNA databases

| miRNA | Predicted target genes |
| --- | --- |
| miR-106b-5p, miR-17-5p | *TGFβR2* |
|  | *SMAD4* |
|  | *RBL2* |
|  | E2F5 |
|  | HBP1 |
|  | KIF23 |
|  | NAGK |
|  | PKD2 |
|  | TNFRSF21 |
|  | ZFYVE26 |
